# VariantValidator: Accurate validation, mapping, and formatting of sequence variation descriptions

**DOI:** 10.1002/humu.23348

**Published:** 2017-10-17

**Authors:** Peter J. Freeman, Reece K. Hart, Liam J. Gretton, Anthony J. Brookes, Raymond Dalgleish

**Affiliations:** ^1^ Department of Genetics University of Leicester Leicester United Kingdom; ^2^ Invitae Inc. San Francisco California; ^3^ Genome Medical Inc. San Francisco California; ^4^ IT Services University of Leicester Leicester United Kingdom

**Keywords:** HGVS variant nomenclature, reference sequences, sequence variants, sequence variation, validation, variant call format, VCF

## Abstract

The Human Genome Variation Society (HGVS) variant nomenclature is widely used to describe sequence variants in scientific publications, clinical reports, and databases. However, the HGVS recommendations are complex and this often results in inaccurate variant descriptions being reported. The open‐source hgvs Python package (https://github.com/biocommons/hgvs) provides a programmatic interface for parsing, manipulating, formatting, and validating of variants according to the HGVS recommendations, but does not provide a user‐friendly Web interface. We have developed a Web‐based variant validation tool, VariantValidator (https://variantvalidator.org/), which utilizes the hgvs Python package and provides additional functionality to assist users who wish to accurately describe and report sequence‐level variations that are compliant with the HGVS recommendations. VariantValidator was designed to ensure that users are guided through the intricacies of the HGVS nomenclature, for example, if the user makes a mistake, VariantValidator automatically corrects the mistake if it can, or provides helpful guidance if it cannot. In addition, VariantValidator has the facility to interconvert genomic variant descriptions in HGVS and Variant Call Format with a degree of accuracy that surpasses most competing solutions.

## 1 INTRODUCTION

The Human Genome Variation Society (HGVS) nomenclature for the description of human sequence variants (den Dunnen et al., 2016) is widely adopted by scientific journals and variant databases and is endorsed by professional organizations (Deans, Fairley, den Dunnen, & Clark, 2016; Richards et al., 2015; Tack, Deans, Wolstenhome, Patton, & Dequeker, 2016). As high‐throughput sequencing has become more common, HGVS recommendations have evolved to communicate a plethora of new variants to the scientific and healthcare communities (Taschner & den Dunnen, 2011). This has resulted in some aspects of the nomenclature being somewhat difficult to comprehend and use, for experts and non‐experts alike, and so has resulted in many instances of inaccurate communication of variant data. Consequently, high‐quality user‐friendly tools are required to help investigators validate variant descriptions to ensure that the described variant is valid and consistent with the predicted phenotypic effect. There is also a need for high‐quality tools that can convert high‐throughput sequence variation descriptions (e.g., the Variant Call Format [VCF] https://github.com/samtools/hts-specs) (Danecek et al., 2011) into accurate descriptions of each variant using HGVS nomenclature with respect to all relevant reference sequences (i.e., genomic reference sequences and transcript reference sequences), and vice versa.

Mutalyzer (Wildeman, van Ophuizen, den Dunnen, & Taschner, 2008) already provides a Web interface for constructing, validating, and transforming sequence variant descriptions. Mutalyzer is a powerful and widely used tool but it cannot comprehensively validate all variants that comply with the HGVS nomenclature. For example, its primary Name Checker interface cannot validate sequence variants described in the context of chromosomal DNA reference sequences, for example, NC_000017.10:g.48275363C>A (GRCh37). Mutalyzer's Position Converter will accept NC_000017.10:g.48275363C>A and map the variant base position to relevant transcript descriptions. However, it does not attempt to validate the stated reference base against the reference sequence, that is, it will accept the syntactically correct, but data‐incorrect, variant description NC_000017.10:g.48275363G>A (rather than C>A) and map it to NM_000088.3:c.589C>T rather than G>T. In addition, Mutalyzer lacks the ability to validate intronic variants with respect to coding sequence (transcript) reference sequences, for example, NM_000088.3:c.589‐1G>T, because it lacks the facility to map transcripts to the genome and back again.

The Ensembl Variant Effect Predictor (VEP) (McLaren et al., 2016) provides much of the necessary functionality required to map sequence variant descriptions in the VCF format to the HGVS format. However, VEP currently cannot handle sequence mismatches that often occur between genome reference sequences and the corresponding aligned RefSeq transcript sequences, for example, chromosomal variant NC_000001.11:g.216046439A>C (GRCh37) returns the HGVS transcript description NM_206933.2:c.6317T>G and predicted protein description NP_996816.2:p.Ile2106Arg, whereas the valid descriptions should be NM_206933.2:c.6317C>G and NP_996816.2:p.(Thr2106Arg). VEP will therefore return inaccurate transcript and protein HGVS descriptions when there exist sequence discrepancies between the transcript and genomic sequences. VEP is also currently unable to validate some of the syntactically simple HGVS description types, for example, inversions, such as NM_000088.3:c.591_593inv.

The hgvs Python package (Hart et al., 2015) has several distinct advantages over Mutalyzer: (a) variants are validated in the context of the specified reference sequence; (b) intronic variants, with respect to coding sequences, can be mapped to corresponding chromosomal sequences and the reference base validated at the chromosomal sequence level; (c) tools are provided to map variation within one transcript to other transcripts that overlap the same genomic coordinates. However, the hgvs Python package does not provide a user‐friendly interface, and it does not currently possess the functionality to process non‐HGVS formatted variant descriptions such as VCF (Danecek et al., 2011) or the pseudo‐VCF format (e.g., 11‐5248232‐T‐A), which is used by ExAC (https://exac.broadinstitute.org/) (Lek et al., 2016), VarSome (https://varsome.com/), and other related resources.

We have built a simple and intuitive Web interface, VariantValidator (https://variantvalidator.org/), which harnesses and automates the key components of the hgvs Python package (Hart et al., 2015). We have also incorporated additional functionality such that VariantValidator is able to accurately map between the HGVS and VCF sequence variation description formats, enabling rapid transformation of high‐throughput sequence data generation into HGVS‐compliant variant descriptions. Unlike any of the aforementioned tools, VariantValidator adheres fully to the current HGVS guidelines in regard to the description of intronic variants with respect to a transcript reference sequence. VariantValidator produces complete descriptions in the format <genomic reference sequence> (<transcript reference sequence>):c.<position> <observed variation>; for example, NG_007400.1(NM_000088.3):c.589‐1G>T (http://varnomen.hgvs.org/bg-material/refseq/). Finally, VariantValidator has been designed to provide users with informative guidance relating to any variant‐description errors, which may have been detected, rather than terse error messages.

## 2 METHODS

The VariantValidator interface is deployed on an Apache 2.0 HTTP server using mod_wsgi (https://github.com/GrahamDumpleton/mod_wsgi) and is written in Python using the Flask micro‐framework (http://flask.pocoo.org/). At the time of writing, VariantValidator is limited to the functionality provided in hgvs Python package version 1.0.0a1 (https://github.com/biocommons/hgvs) and Universal Transcript Archive (UTA) version uta_20170707 (https://github.com/biocommons/uta/). To ensure optimal performance, VariantValidator benefits from a local installation of uta_20170707 and is programmed such that sequences (nucleotide or amino acid) are recovered from a locally installed version of SeqRepo (https://github.com/biocommons/biocommons.seqrepo). VariantValidator uses several MySQL look‐up tables containing: (a) RefSeq Transcript IDs (NM_ or NR_), current transcript name, HGNC gene symbol, corresponding gene symbol used by UTA (e.g., the previous UTA build (uta_20170117) used the gene symbol *LEPRE1*, which was actually updated by HGNC to *P3H1* in December 2014); (b) coordinate‐based mappings (chromosome number, start position, end position) of RefSeqGene (NG_) sequences to chromosomal sequences (NC_) for both genome builds GRCh37 and GRCh38; (c) LRG reference sequence IDs and LRG transcript reference sequence IDs with their associated RefSeqGene IDs or RefSeq transcript IDs respectively; (d) and RefSeq Transcript ID, HGNC ID, current gene symbol, current gene name, and coordinate‐based mappings of the transcripts to genome builds GRCh37 and GRCh38. The lookup tables were compiled and are updated on a monthly basis by custom Python scripts. The required data are downloaded directly from NCBI (https://ftp.ncbi.nih.gov/refseq/H_sapiens/), HGNC (http://ftp.ebi.ac.uk/pub/databases/genenames/new/tsv/hgnc_complete_set.txt) and UCSC (https://genome.ucsc.edu/cgi-bin/hgTables).

The most significant current limitations of the hgvs Python package at this stage are: (a) some classes of complex variation, for example, predicting the effect at the protein level of nucleotide inversions (however, we have incorporated supporting code into VariantValidator to overcome this issue); and (b) some RefSeqGene sequences and historic versions of some transcripts are either absent from UTA, or they are not mapped to a particular genome build (most usually GRCh37). This is a result of relevant historical mapping datasets being unavailable from NCBI for these sequences. However, significant efforts are being made to develop a robust protocol to map the absent sequences accurately to genome builds GRCh37 and GRCh38.

VariantValidator has some limitations in that it does not yet implement the full functionality of the hgvs Python package. For example, there is no support for the output of variant descriptions in the context of Ensembl transcripts (Aken et al., 2017) using the Genebuild alignment method. This decision is based on the HGVS variant nomenclature recommending the use of NCBI RefSeq (O'Leary et al., 2016) and LRG (Dalgleish et al., 2010; MacArthur et al., 2014) reference sequences (http://varnomen.hgvs.org/bg-material/refseq/). However, VariantValidator will accept the input of variant descriptions with respect to Ensembl transcript sequences if a sequence‐identical RefSeq transcript reference sequence is available. Requests to implement missing functionality and bug reports can be submitted to the VariantValidator administrator by clicking the “Contact admin” link at the top right corner of the Web page. In addition, if errors are detected during the processing of input variation descriptions, VariantValidator automatically contacts admin via email and the associated bug is quickly rectified.

## 3 RESULTS AND DISCUSSION

### 3.1 What is the purpose of VariantValidator?

The hgvs Python package is a powerful tool for: (a) validating HGVS variant descriptions; (b) mapping variation between different reference sequence types (e.g., chromosome, gene and transcript reference sequences); (c) formatting (i.e., normalizing or shuffling) descriptions of variants in stretches of repetitive sequence so that they fully comply with HGVS nomenclature guidelines. Although the hgvs Python package is intended as a foundation for tool developers, many users would prefer to access it through a simple and intuitive Graphical User Interface (GUI). VariantValidator provides a Web‐based GUI for the hgvs Python package.

During the development of VariantValidator we set several key objectives: (a) the interface must be clean, concise, and easy to read; (b) the application must guide users in HGVS nomenclature compliance, providing clear recommendations, prompts, and warnings where required; (c) mapping variants between reference sequences should be automated and the results displayed to the user on a single clearly laid out Web page; (d) the application must provide additional features that are unavailable in the hgvs Python package so that the user is provided with a wide range of useful information; for example, the conversion of c. HGVS variant descriptions into the VCF format, up to date HGNC gene symbols (Yates et al., 2017) and transcript descriptions; (e) links that enable the user to access relevant external data, for example, NCBI RefSeq records and aggregated data resources, for example, VarSome (https://varsome.com/) (as discussed below).

VariantValidator provides users with an alternative to the commonly used Mutalyzer Web‐based software. It was specifically designed to provide users with functionality that Mutalyzer is unable to provide, including: (a) automated validation of intronic variants with respect to transcript reference sequences and assembly of HGVS‐compliant intronic c. variant descriptions; (b) the ability to appropriately re‐format specific variant descriptions such that they cross into intronic sequence, and remain fully compliant with HGVS recommendations; (c) the ability to map sequence variation at the chromosomal level to all relevant transcripts; (d) the ability to accept and process non‐HGVS variant descriptions such as VCF and hybrid HGVS:VCF variant descriptions; (e) helpful warnings and, where appropriate, automated “hand‐holding,” which guides the user through the complexities of the HGVS nomenclature. These functions are discussed below. In addition to the standard batch validation tool in VariantValidator, there is also a tool that converts variant data from VCF files and feeds them directly into the batch validation tool. We currently implement a fair usage policy limiting the batch tools toward processing 20,000 variants in a single job. However, we are in the process of streamlining the batch tool and intend to relax this restriction as soon as possible.

### 3.2 The VariantValidator interface

**Figure 1:**
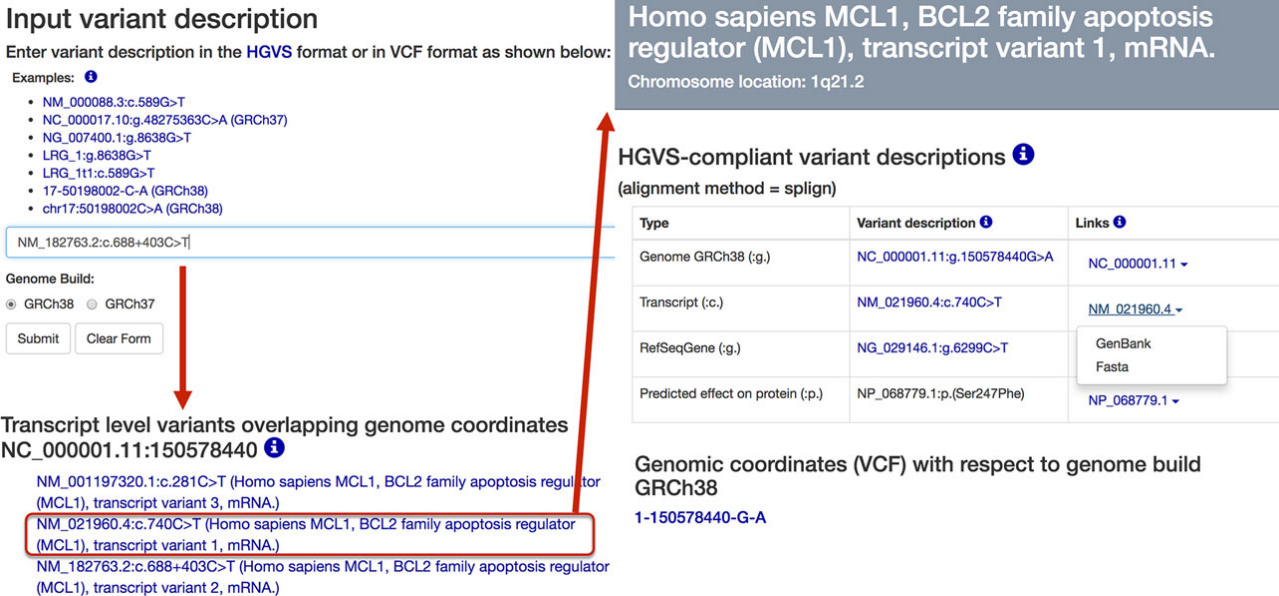
Mapping of variants onto alternative transcripts. Submitted variant descriptions are automatically mapped, via the selected genome build (GRCh38), onto all other transcripts that overlap the same genomic position. In this example, NM_182763.2:c.688+403C>T, which is intronic with respect to *MCL1* transcript variant 2 mRNA, is mapped to an exonic variant in *MCL1* transcript variant 1 mRNA, NM_021960.4:c.740C>T. The same initial variant description also maps to an exonic variant in *MCL1* transcript variant 3 mRNA, NM_001197320.1:c.281C>T

VariantValidator provides an interface allowing validation of genomic variants (e.g., NC_000001.10:g.150550916G>A or NG_029146.1:g.6299C>T) or transcript variants (e.g., NM_182763.2:c.688+403C>T). It can also validate variant descriptions in VCF‐like (pseudo‐VCF) formats such as 1‐150550916‐G‐A or 1:150550916G>A (GRCh37); and unofficial “hybrid” HGVS:VCF formats (e.g., NC_000016.9:g.2099572TC>T is corrected to NC_000016.9:g.2099575delC). As an illustration, consider the variant in the *MCL1* gene, NM_182763.2:c.688+403C>T, used as an example in the original description of the hgvs Python package (Hart et al., 2015). This can be submitted to VariantValidator as follows: (a) type or paste the variant description into the input text box; (b) select a genome build; (c) click Submit (Figure 1). On submission, the input variant description is validated to ensure that: (a) it complies with the HGVS recommendations; (b) the reported nucleotide sequence alterations (e.g., deletions, duplications, substitutions, etc.) are consistent with the reference sequence; (c) intron/exon boundary coordinates are correct. HGVS‐compliant variant descriptions are then presented to the user in the context of all available corresponding reference sequences. In addition, we have incorporated functions to return data on gene‐level variant descriptions (RefSeqGene) and chromosomal locations in both HGVS format and a commonly used VCF‐like format. If validation fails, an error message, including the reason for failure, is returned with guidance to the user.

The UTA (https://github.com/biocommons/uta) (Hart et al., 2015) stores sequence alignment data accessed by hgvs but does not provide the descriptive name of transcript reference sequences, for example, Homo sapiens MCL1, BCL2 family apoptosis regulator (MCL1), transcript variant 1, mRNA. Providing this name is particularly useful if a gene (e.g., MCL1) encodes more than one transcript variant because, unlike the reference sequence ID (NM_021960.4), the name clearly identifies the transcript variant (transcript variant 1) against which variation is being reported. VariantValidator stores regularly updated RefSeq data (see *Methods*) and displays the corresponding descriptions of transcript reference sequences. VariantValidator determines that NM_182763.2:c.688+403C>T refers to an intronic variant in “Homo sapiens BCL2 family apoptosis regulator (MCL1), transcript variant 2, mRNA.” Three well‐characterized RefSeq transcripts exist for the *MCL1* gene and VariantValidator automatically maps the submitted variant to its genomic coordinates and also to any other transcripts that overlap the genomic coordinates specified in the variant description. As shown in Figure 1, we have mapped an intronic variant with respect to *MCL1* transcript variant 2 mRNA to an exonic variant in *MCL1* transcript variant 1 mRNA, NM_021960.4:c.740C>T. The same initial variant description also maps to an exonic variant in *MCL1* transcript variant 3 mRNA, NM_001197320.1:c.281C>T. VariantValidator provides complete HGVS‐compliant variant descriptions detailing how sequence variation at the genomic level maps to sequence variation in each overlapping transcript. VariantValidator also automatically provides the user with a pseudo‐VCF description of the input variant description (1‐150550916‐G‐A, GRCh37 or 1‐150578440‐G‐A, GRCh38—Figure 1), which enables the user to query external data resources, for example, VarSome. If VariantValidator is unable to recover necessary information relating to a specific transcript from UTA, for example, if the previous version of an updated reference sequence is not mapped to the supported genome builds (GRCh37 and GRCh38), reference sequences that are actually supported in UTA can be identified using the reference sequence finder (https://variantvalidator.org/ref_finder/).

VariantValidator provides links to RefSeq sequence records (Figure 1) and links to VarSome.com (https://varsome.com/), which provides aggregated information on the input variant description.

### 3.3 Ease of use

We aim to consistently use simple workflows, for example, a three‐click workflow that allows a genomic variant correctly mapping to genome build GRCh37 (NC_000001.10:g.150550916G>A or 1‐150550916‐G‐A) to be projected via a transcript level variant description (NM_182763.2:c.688+403C>T) onto genome build GRCh38 (NC_000001.11:g.150578440G>A or 1‐150578440‐G‐A). This feature is particularly useful for transforming variant descriptions from genomic data, in the VCF format, into the HGVS format for publication and use in clinical reports and databases. Similarly, this workflow can be used to project the data stored in publications, clinical reports, and databases onto the two most recent genome builds (GRCh37 and GRCh38). In addition, we have incorporated various subroutines to assist inexperienced HGVS nomenclature users. For example, when validating incorrectly reported intron/exon boundary coordinates (e.g., NM_182763.2:c.687+404C>T), VariantValidator performs automatic re‐mapping to the nearest exon boundary and displays the most probable valid variant description to the user (i.e., NM_182763.2:c.688+403C>T).

**Figure 2:**
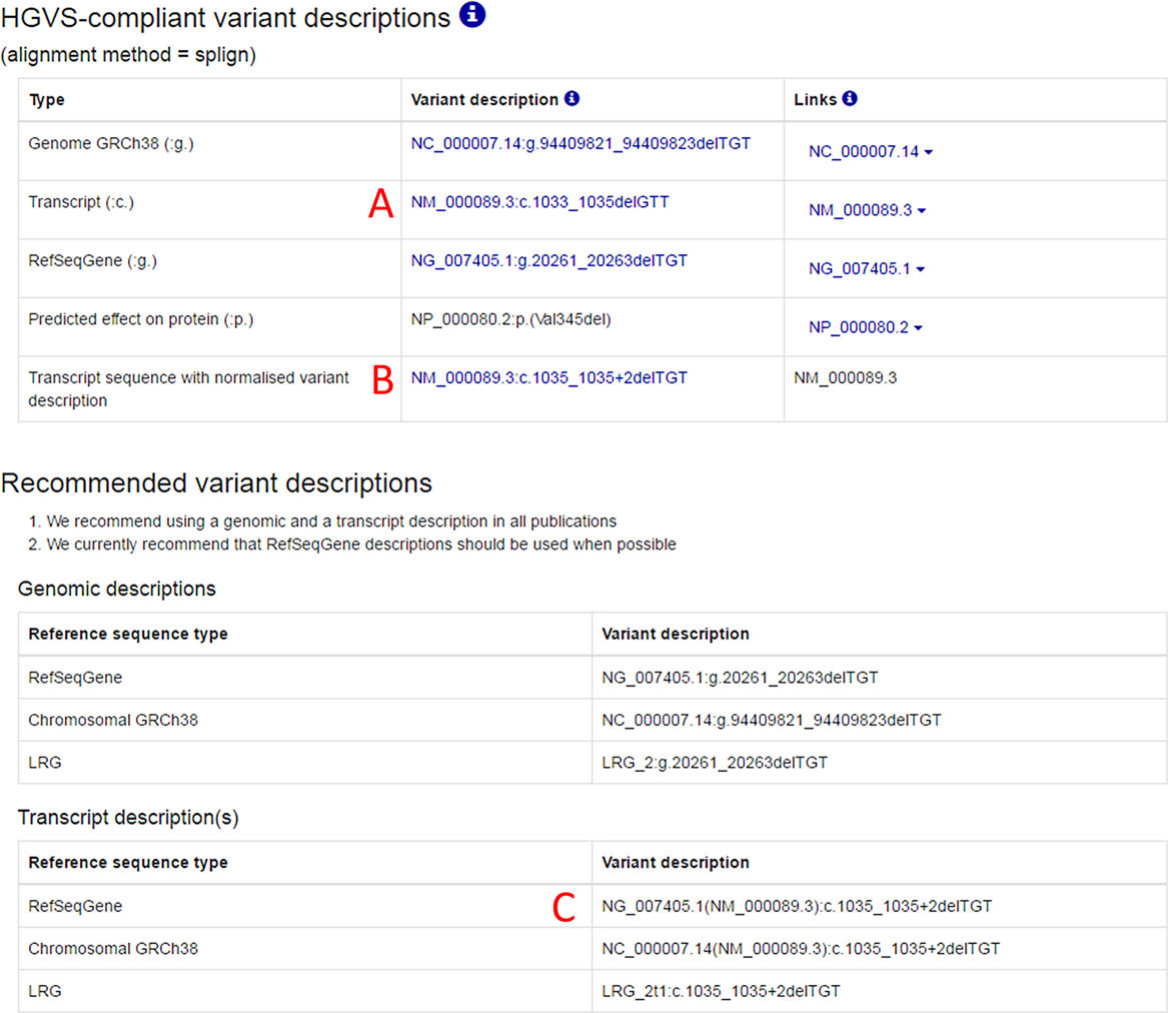
Variant descriptions at exon/intron boundaries. This illustrates how a three‐base deletion in the *COL1A2* gene at the junction of the 3′ end of exon 19 with the adjacent intron might be described in two different ways in the context of the RefSeq transcript reference sequence NM_000089.3. Description A shows that the three deleted bases can be described at position NM_000089.3:c.1033_1035 where the deleted bases are GTT, but Description B shows that the variant can be normalized and described at position NM_000089.3:c.1035_1035+2 where the deleted bases are TGT. The latter description corresponds with the genomic variant description NC_000007.13:g.94039133_94039135delTGT. Formally, intronic variants described in the context of a transcript reference sequence must be accompanied by a genomic reference sequence to allow full verification of the variant. This is illustrated by Description C

VariantValidator has additional advantages over Mutalyzer. These include: (a) the use of the non‐coding variant type (n.) is fully compliant with HGVS recommendations; and (b) variant descriptions and alignments are provided for both coding and genomic sequences thus allowing the user to make informed decisions when validating complex variants, such as those close to exon/intron boundaries. To emphasize these two points, if the variant description NM_000089.3:c.1033_1035del (Molyneux, Starman, Byers, & Dalgleish, 1993) is submitted to Mutalyzer, it returns the original variant description and NM_000089.3:n.1504_1506del, which is a description relative to the first base of the transcript. Although Mutalyzer informs the user that this description is “Not for use in LSDBs in case of protein‐coding transcripts” it gives the misleading impression that the description could be valid and HGVS‐compliant in other contexts. In contrast, VariantValidator returns (among others) the original variant description NM_000089.3:c.1033_1035delGTT, the corresponding genome variant description NC_000007.13:g.94039133_94039135delTGT (GRCh37) and a normalized (3´‐shuffled) coding variant description that maps the deletion across an exon/intron boundary relative to the genomic DNA sequence onto which the transcript maps, NM_000089.3:c.1035_1035+2delTGT (Figure 2).

During development testing of VariantValidator, analysis of error logs indicated that some users were specifying HGNC gene symbols, rather than valid sequence records, as sequence references for variant validation, for example, COL5A1:c.5071A>T. When COL5A1:c.5071A>T is submitted to VariantValidator, the user is warned that “HGVS variant nomenclature does not allow the use of a gene symbol (COL5A1) in place of a valid reference sequence” (http://varnomen.hgvs.org/bg-material/refseq/). VariantValidator then provides the user with valid transcript reference sequences that might be used in the context of this variant to generate a valid description. The user‐corrected variant description must then be submitted to VariantValidator to determine whether or not it is, in fact, valid.

### 3.4 Validation of intronic variation with respect to transcript reference sequences

HGVS nomenclature recommendations provide guidance on how to describe intronic sequence variation with respect to transcript (coding DNA) reference sequences, for example, the imaginary variant NM_012345.6:c.88+2T>G. Indeed, the guidelines acknowledge that describing intronic splice site variants in this way provides an immediate indication of the location of the variant. However, since RefSeq transcript reference sequences do not contain intronic sequence, it is impossible to directly verify in this example description that the reference base at the +2 position is actually a T.

The practicality of validating an intronic variant that is reported in the context of a transcript reference sequence is well illustrated by the sequence variant NM_022356.3:c.2055+18G>A (Willaert et al., 2009). Mutalyzer cannot validate the variant description in this format even though it is compliant with the HGVS nomenclature. The solution is to specify the variant in the context of the corresponding RefSeqGene reference sequence, NG_008123.1, but there are three transcript variants (NM_022356.3, NM_001146289.1, and NM_001243246.1.) for the *P3H1* gene encoding protein isoforms, which differ at their C‐terminal ends. Mutalyzer warns the user that three transcripts are found for the gene and asks the user to select one of the three from “001, 002, 003.″ The user must then compile a new variant description that specifies the transcript to which the variant description refers. For the example *P3H1* variant, the required working description for Mutalyzer becomes NG_008123.1(LEPRE1_v003):c.2055+18G>A. Two issues should be noted. The cached version of NG_008123.1 at Mutalyzer, at the time of writing, is date‐stamped 29‐May‐2014, which predates the change of gene symbol for this gene by HGNC from *LEPRE1* to *P3H1* in December 2014. This explains why Mutalyzer designates the three transcripts as LEPRE1_v001, LEPRE1_v002, and LEPRE1_v003 rather than as P3H1_v001, P3H1_v002, and P3H1_v003. The more confusing aspect of this scheme is that the numeric parts of these three transcript designations do not correspond with the transcript variant numbers assigned by RefSeq to the three transcript sequences (Table 1). Instead, they are derived from the sequential order in which the three transcript variants are annotated in the RefSeqGene record NG_008123.1. VariantValidator does, however, provide support for users who attempt to validate variant descriptions in the formats similar to NG_008123.1(LEPRE1_v003):c.2055+18G>A or NG_008123.1:c.2055+18G>A where a RefSeqGene reference sequence is being used inappropriately for c. positions that should be annotated with respect to a coding‐DNA sequence. When NG_008123.1(LEPRE1_v003):c.2055+18G>A is submitted, VariantValidator warns “NG_:c.PositionVariation descriptions should not be used unless a transcript reference sequence has also been provided e.g. NG_(NM_):c.PositionVariation: For additional assistance, submit NG_008123.1:c.2055+18G>A to VariantValidator.” When NG_008123.1:c.2055+18G>A is submitted, VariantValidator automatically provides the user with the available transcripts to which the original variant description may have been mapped (e.g., NG_008123.1(NM_022356.3):c.2055+18G>A (Homo sapiens prolyl 3‐hydroxylase 1 (P3H1), transcript variant 1, mRNA.), with the caveat that the provided variant description must be subsequently submitted to VariantValidator to determine whether it is valid. Describing an intronic sequence variant as NG_008123.1(NM_022356.3):c.2055+18G>A makes good practical sense. It reveals that the variant is intronic (c.f. NG_008123.1:g.24831G>A) and defines both the RefSeqGene and RefSeq sequences in the context of which the variant is described. In contrast, the Mutalyzer transcript designation (LEPRE1_003, or P3H1_003) coveys no absolute (or immediately understandable) identifier for the transcript sequence. Describing variants in the joint context of a transcript and genomic reference sequences is a feature of HGVS nomenclature version 15.11. There will be instances where no RefSeqGene reference exists for a gene, for example, *HOXD12*, at the time of writing, but intronic variants can be validly described for such genes in the context of genome and transcript reference sequences, for example, NC_000002.11(NM_021193.3):c.574+1G>A (http://varnomen.hgvs.org/bg-material/refseq/). VariantValidator uses this convention when recommending variant descriptions with the caveat that if a RefSeqGene sequence record is available, it should take precedence over the chromosomal record. Mutalyzer does not adopt a fallback position for describing intronic variation when there is no RefSeqGene record, thus it cannot describe or validate intronic sequence variants for approximately 75% of genes with protein‐coding or non‐coding transcripts. Not only does VariantValidator use the chromosomal sequence as its reporting fallback position, it also clearly states when RefSeqGene records are not available.

**Table 1:** Mutalyzer transcript designations do not correspond with the RefSeq transcript sequence definitions

RefSeq accessions and versions for *P3H1* gene transcripts	RefSeq sequence definitions	Mutalyzer transcript designations
NM_022356.3	Homo sapiens prolyl 3‐hydroxylase 1 (P3H1), transcript variant 1, mRNA.	LEPRE1_v003
NM_001146289.1	Homo sapiens prolyl 3‐hydroxylase 1 (P3H1), transcript variant 2, mRNA.	LEPRE1_v002
NM_001243246.1	Homo sapiens prolyl 3‐hydroxylase 1 (P3H1), transcript variant 3, mRNA.	LEPRE1_v001

### 3.5 Validation and mapping of chromosomal variants to underlying genes and transcripts

In contrast to Mutalyzer Name Checker (https://mutalyzer.nl/name-checker), VariantValidator will validate variant descriptions with respect to chromosomal reference sequences, for example, NC_000017.10:g.48275363C>A or 17‐48275363‐C‐A (GRCh37), and is particularly useful when validating descriptions in VCF files generated by NGS data analysis programs. This allows users to accurately map a chromosomal sequence variant to all overlapping genes and transcripts and automatically generate fully HGVS‐compliant variant descriptions with respect to each. The Mutalyzer Position Converter does perform a similar function but does not actually validate the input variant description, meaning that mistyped descriptions are not identified. In addition, the Position Converter can produce variant mappings that report positions that lie distant from the reference sequences in the context of which they are reported. For example, the *COL1A1* variant NC_000017.10:g.48275363C>A is correctly mapped by Position Converter to *COL1A1* gene and transcript records, but also to LOC1005065: XR_109403.1:n.570‐4413C>A. There is no gene corresponding to LOC1005065 and XR_109403.1 is a retired sequence that corresponds to the former predicted locus LOC100506522. VariantValidator only maps variants to locations in fully validated RefSeq transcripts for which there is supporting biological evidence (NM_ and NR_) genes (NG_) and chromosomes (NC_), which are included in the UTA.

### 3.6 Features in VariantValidator that are additional to the underlying hgvs Python package

The VariantValidator Web interface provides simultaneous automated validation and mapping to all relevant reference sequences and displays the data in a single view. This cannot be achieved using hgvs from the programming interface, so VariantValidator provides a level of functionality and detail to users that hgvs alone cannot provide.

VariantValidator provides users with additional functionality, not discussed above, that enhances the outputs that can be achieved using hgvs alone. For example, to ensure that variants can be validated in the context of recently created RefSeqGene sequences, VariantValidator retrieves RefSeqGene records, where possible, that are not present in the UTA database. It then maps sequence variation with respect to coding reference sequences via chromosomal coordinates to the RefSeqGene coordinates. Similarly, VariantValidator uses lookup tables to match LRG and LRG transcript reference sequences with their corresponding RefSeqGene and RefSeq transcript sequences respectively, thus VariantValidator provides support for users wishing to validate variant descriptions with respect to LRG reference sequences. Similar logic is applied for users wishing to input variant descriptions with respect to Ensembl transcript reference sequences with the caveat that VariantValidator will not output variant descriptions relating to Ensembl transcript reference sequences.

The ability to format intronic variant descriptions in the form NG_008123.1(NM_022356.3):c.2055+18G>A is not intrinsic to the hgvs Python package and has been developed specifically for VariantValidator. This is also true of the ability to map inversions within coding reference sequences to predicted protein sequence variation. This requires extracting the coding sequence, generating a variant coding sequence, translating both sequences, and finally comparing the protein sequences to extract a description of the predicted protein sequence variation. We have also developed some of the basic VariantValidator‐specific functions to provide additional features such as: (a) the generation of pseudo‐VCF format variant descriptions; (b) integration of output from VarSome.com, which provides an alignment tool that displays variation at the sequence level along with aggregated data relevant to the submitted variant; (c) tools to extract variant data from VCF files and pseudo‐VCF variant descriptions and re‐format the VCF calls into variant descriptions that can be handled by the hgvs Python package; (d) a fully automated batch validation tool; and (e) VariantValidator generates a series of custom error messages such that users are informed that VariantValidator automatically corrects errors made by the user when it is able to do so, or provide informative information such that the user can correct their own mistakes when VariantValidator is unable to do so. These features allow VariantValidator to access and supplement the wide range of tools provided by the hgvs Python package. VariantValidator can, therefore, provide users with a clean, concise and user‐friendly Web interface that enables responsive validation of sequence variants.

### 3.7 Mutalyzer features not supported by VariantValidator

Although VariantValidator offers an alternative to Mutalyzer, it does not yet provide the full range of functionality that Mutalyzer currently offers, for example, a HGVS name generator (https://mutalyzer.nl/name-generator); a description extractor (https://mutalyzer.nl/description-extractor); and a function to convert amino acid substitutions into likely nucleotide substitutions (https://mutalyzer.nl/back-translator).

Although the hgvs Python package functions allow all common variant types to be parsed into the necessary formats to be handled by its functions, a key strength of the package is its ability to map sequence‐level variation between different reference sequences. In the current build of the hgvs Python package (1.0.0a1), two particular variant types are currently not well supported with respect to mapping. Gene conversions can be validated with respect to sequence‐level variation and HGVS compliance. However, they cannot yet be mapped between reference sequences or mapped into theoretical protein sequence variation descriptions. In this respect, VariantValidator is only slightly less capable than Mutalyzer that can validate the syntax of a conversion description (e.g., NM_000088.3:c.4_64conNM_004006.1:c.123_171), but not project the variant to other reference sequence contexts. However, we intend to address this deficiency in a future release of VariantValidator.

### 3.8 Plans for further development

The hgvs Python package and UTA are undergoing continuing development and we may consider expanding VariantValidator to provide support for additional specific types of sequence variation and reference sequence types in the future. Proper future support for inversions might allow us to use native hgvs Python package functions rather than our own custom code. Similarly, support for gene conversions would be a desirable feature. However, the desire to properly support inversions and conversion must be set against the fact that instances of such variant types are relatively rare. We are currently re‐developing our batch analysis tools (batch validator and vcf2hgvs) to enhance their performance so that results are returned to our users more quickly.
